# Wearable EEG Device for Continuous Spike–Wave in Sleep

**DOI:** 10.15844/pedneurbriefs-34-10

**Published:** 2020-12-02

**Authors:** Hirokazu Oguni

**Affiliations:** 1 Department of Pediatrics, Tokyo Women's Medical University, Tokyo, Japan

**Keywords:** ESES, CSWS, Spike Index, Wearable Ambulatory EEG

## Abstract

Investigators from the Hospital Dona Estefânia, Escola Superior de Tecnologias e Saúde de Lisboa and Centro Hospitalar Psiquiátrico de Lisboa investigated 38 patients with continuous spike-wave of sleep (CSWS) syndrome.

Investigators from the Hospital Dona Estefânia, Escola Superior de Tecnologias e Saúde de Lisboa and Centro Hospitalar Psiquiátrico de Lisboa investigated 38 patients with continuous spike-wave of sleep (CSWS) syndrome. A 10-20 montage long-term ambulatory electroencephalography (aEEG) and a wearable aEEG with two bipolar EEG channels simultaneously validate the latter's usefulness to quantify the spike index (SI) to analyze the EEG response to the treatments. The SI was digitally calculated every 10 minutes by semiautomatic template-match-spike search analysis compared to both aEEG. They also compared daily SI stability (three-consecutive night, N=4) and monthly SI stability (N=10) in individual patients and employed the SI obtained from averaging the maximum SI for each of the first four sleep cycles (avSI) for comparison to reduce variability. The wearable aEEG examinations were repeated up to 8 times per patient. The response to therapy using corticosteroids (n=10), sulthiame (n=7), and the ketogenic diet (n=3) was assessed by comparison of the avSI after therapy. As a result, corticosteroids most effectively reduced the SI among therapies, although sizeable individual variability in both the amount and time of onset of clinical responses. In conclusion, the wearable aEEG with two bipolar EEG channels, which are easily attached and tolerable for children, can provide accurate SI quantification repeatedly in clinical settings. [1]

COMMENTARY. CSWS syndrome, also known as encephalopathy related to status epilepticus during slow sleep (ESES), is one of the most common epileptic encephalopathies of childhood [2]. In this syndrome, epileptic seizures themselves may be infrequent or even absent, but EEG demonstrates marked activation of spike-wave discharges during sleep. The latter EEG phenomenon is believed to cause cognitive/behavioral deterioration in this syndrome because it electrically affects cerebral function [2]. As CSWS has been observed before such deteriorations, it is essential to estimate the SI for early therapeutic intervention. In clinical practice, the SI has been evaluated by routine EEG examinations during naps or long-term EEG during hospitalization; however, both methods have limitations, i.e., difficult interpretation of the SI and expense of repeated examinations [3,4]. Thus, it is ideal to evaluate the SI using aEEG for 24 hours at home. However, the aEEG device currently available is expensive, complicated to attach, and also intolerable to wear for a long time for children. In this study, the authors developed the wearable aEEG device capable of recording with bipolar two channels and digitally quantifying the SI, which was demonstrated to be accurate by comparing the SI with that of the full 10-20 long-term aEEG. The semiautomatic template-match-spike search analysis they employed can plot the SI every 10 minutes during overnight sleep accurately and without difficulty [5]. In CSWS syndrome, the methods to determine the SI and its boundary to limit the CSWS definition (initially SI >85%) have remained major concerns among specialists [2-4]. In terms of its simplicity, cost, and unification of the methods to quantify the SI, the wearable aEEG in this study should be useful not only in clinical practice but also in CSWS syndrome studies.

## Disclosures

The author has declared that no competing interests exist.
